# Role of Intravitreal Antivascular Endothelial Growth Factor Injections for Choroidal Neovascularization due to Choroidal Osteoma

**DOI:** 10.1155/2014/210458

**Published:** 2014-07-23

**Authors:** Ahmad M. Mansour, J. Fernando Arevalo, Eman Al Kahtani, Hernando Zegarra, Emad Abboud, Rajiv Anand, Hamid Ahmadieh, Robert A. Sisk, Salman Mirza, Samuray Tuncer, Amparo Navea Tejerina, Jorge Mataix, Francisco J. Ascaso, Jose S. Pulido, Rainer Guthoff, Winfried Goebel, Young Jung Roh, Alay S. Banker, Ronald C. Gentile, Isabel Alonso Martinez, Rodney Morris, Neeraj Panday, Park Jung Min, Emilie Mercé, Timothy Y. Y. Lai, Vicky Massoud, Nicola G. Ghazi

**Affiliations:** ^1^Departments of Ophthalmology, American University of Beirut, Rafic Hariri University Hospital, P.O. Box 113-6044, Beirut, Lebanon; ^2^Vitreoretinal Division, The King Khaled Eye Specialist Hospital, Riyadh, Saudi Arabia; ^3^Wilmer Eye Institute, The Johns Hopkins University, Baltimore, MD 21287, USA; ^4^Retina Associates of Cleveland, 3401 Enterprise Parkway, Suite 300, Beachwood, OH 44122, USA; ^5^Texas Retina Associates, University of Texas Southwestern Medical Center, Dallas, TX 75231, USA; ^6^Ophthalmic Research Center, Shahid Beheshti University of Medical Sciences, Labbafinejad Medical Center, Tehran, Iran; ^7^Cincinnati Eye Institute, Cincinnati, OH 45212, USA; ^8^Birmingham Midland Eye Centre, Dudley Road, Birmingham B187QH, UK; ^9^Department of Ophthalmology, Istanbul Faculty of Medicine, Istanbul University, Sergul Sokak Sarali Sitesi, A2 3 Gayrettepe, 34349 Istanbul, Turkey; ^10^Fundación Oftalmológica del Mediterráneo, 46015 Valencia, Spain; ^11^Department of Ophthalmology, “Lozano Blesa” University Clinic Hospital, School of Medicine, University of Zaragoza, 50001 Zaragoza, Spain; ^12^Mayo Clinic, 200 1st Avenue South West Rochester, Minnesota, MN 55902, USA; ^13^University-Eye Hospital Duesseldorf, Moorenstraße 5, 40225 Duesseldorf, Germany; ^14^Department of Ophthalmology, University of Wuerzburg, 97080 Wuerzburg, Germany; ^15^Department of Ophthalmology, St. Mary's Hospital, Catholic University of Korea, Seoul 137-701, Republic of Korea; ^16^Banker's Retina Clinic and Laser Centre, 5 Subhash Society, Behind Ishvar Bhuvan, Ahmedabad, Gujarat 380009, India; ^17^Department of Ophthalmology, New York Eye and Ear Infirmary, New York, NY 10014, USA; ^18^Martinez Center, Avenida María Guerrero 144, Leganés Norte, 28919 Madrid, Spain; ^19^Department of Retina and Vitreous, Aravind Eye Hospital and Postgraduate Institute of Ophthalmology, Avinashi Road, Coimbatore, Tamil Nadu 641014, India; ^20^MM Joshi Eye Institute, Gokul Road, Hosur, Hubli, Karnataka 580021, India; ^21^Maryknoll Medical Center, 4-12 Daecheong-dong, Jung-gu, Busan 600-730, Republic of Korea; ^22^Service d'Ophtalmologie, CHU Dupuytren, 87042 Limoges, France; ^23^Department of Ophthalmology and Visual Sciences, The Chinese University of Hong Kong, Hong Kong

## Abstract

We treated 26 eyes of 25 young patients having a mean age of 30 years with intravitreal vascular endothelial growth factor (VEGF) inhibitor for choroidal new vessel (CNV) formation overlying choroidal osteoma over a mean follow-up of 26 months. Mean number of injections was 2.4 at 6 months, 3.2 at 12 months, and 5.5 at 24 months. CNV was subfoveal in 14 eyes, juxtafoveal in 5, extrafoveal in 5, and peripapillary in 2. By paired comparison, mean decrease from baseline was 119.7 microns at 6 months (*n* = 15; *P* = 0.001), 105.3 microns at 1 year (*n* = 10; *P* = 0.03), and 157.6 microns at 2 years (*n* = 7; *P* = 0.08). BCVA improved by 3.3 lines at 6 months after therapy (*n* = 26; *P* < 0.001), 2.8 lines (*n* = 20; *P* = 0.01) at 1 year, and 3.1 lines (*n* = 13; *P* = 0.049) at 2 years. We conclude that intravitreal anti-VEGF injections improve vision in majority of eyes with CNV from choroidal osteoma.

## 1. Introduction

Choroidal osteoma is a rare ossifying choroidal tumor involving mostly young healthy females in the second decade of life [1–6]. The benign mass appears as a deep yellowish lesion with distinct geographic borders at the juxtapapillary or macular region, with branching “spider” vessels on its surface. The diagnosis is confirmed with the presence of calcification on ultrasonography and computed tomography. Vision is often compromised by gradual atrophy of the overlying retina [6] or by accumulation of either subretinal fluid or subretinal hemorrhage with or without choroidal neovascularisation (CNV). Frequent exams are recommended for patients with choroidal osteoma for early detection of a subretinal neovascular membrane and potential treatment. Therapies have included laser photocoagulation [7, 8], excision of CNV [9], photodynamic therapy (PDT) [10–14], and transpupillary thermal therapy (TTT) [15, 16].

We evaluated clinically and by optical coherence tomography (OCT) [6, 17] the role of intravitreal injections of vascular endothelial growth factor (VEGF) antagonist in the therapy of CNV in choroidal osteomas after their use in some case reports [18–29].

## 2. Materials and Methods

We reviewed retrospectively the files of subjects having choroidal osteoma who were treated with intravitreal injections of bevacizumab or ranibizumab for active CNV. Intravitreal injections of 0.05 mL or 0.1 mL of either bevacizumab (25 mg/mL) or ranibizumab (10 mg/mL) were administered in the office as 3 initial consecutive doses or based on OCT response depending on physician preference. Intravitreal injection was performed using a 30-gauge needle in a sterile manner after topical anesthesia and povidone instillation in the lower conjunctival sac. Bevacizumab (Avastin, Genentech Inc, San Francisco, CA) aliquots were prepared in the hospital pharmacies of the corresponding institutions. Ranibizumab (Lucentis, Genentech Inc, San Francisco, CA) was also used in some centers. A standardized spreadsheet was used to collect the clinical data. Photodynamic therapy (PDT) with intravenous verteporfin (standard dose 6 mg/m^2^ body surface area or half the standard dose) (Visudyne, Novartis AG, Basel, Switzerland) was administered simultaneously in some patients based solely on individual physician preference. Institutional review board/ethics committee approval and patients' signed informed consents were obtained for this study. In addition, this study has been performed in accordance with the ethical standards laid down in the 1964 Declaration of Helsinki for research involving human subjects. The offlabel use of both drugs and their potential risks and benefits was discussed extensively with all patients.

Best corrected visual acuity (BCVA) was assessed using either ETDRS or Snellen charts and listed as logarithm of the minimum angle of resolution (logMar) equivalents. Retreatment was done when there was recurrent activity evaluated by fundus examination, fluorescein angiography (leakage, growth of CNV), or optical coherence tomography. Differences between final and initial BCVA were tested using paired Student *t*-test. Improvement of visual acuity was defined as any fraction of a line of improvement on the ETDRS chart. We did not compare the initial to the posttreatment central foveal thickness because of the different OCT machines among centers as well as because of the need for thickness analysis by gender, race, age, and refractive status [17]. We analyzed only the absolute decrease in central foveal thickness. One patient had bilateral osteoma with CNV arising in one eye and several years later in the fellow eye and hence both eyes were included in the statistical analysis. Significance was set at the 0.05 level. We used SPSS 19 version for statistical calculation (IBM, Armonk, New York, 2010). Literature review till April 2014 (using both PubMed and Google Scholar) was added to ascertain the visual results in this rare disease entity with bevacizumab or ranibizumab therapies [18–29]. Collaborators and one of us (AMM) measured the CNV size on digital fluorescein transit films and the osteoma basal diameter on color films in reference to the horizontal disc diameter by using a transparent reticule or ruler on the computer screen.

## 3. Results

We treated 26 eyes from 25 patients with a mean age at presentation of 29 years (range 8–57 years) with 16 women and 9 men having the following racial distribution: 18 Caucasians, 4 Indians, and 3 Asians. Laterality included 15 right eyes (57.7%) and 11 left eyes (42.3%) (Table 1). Mean follow-up was 26 months (range 6–71 months, median 20 months). The longest osteoma basal diameter varied from 1 to 10 disc diameters with a mean of 4.6 disc diameters. Bevacizumab was injected in 17 eyes (65.4%), ranibizumab in 5 eyes (19.2%), and a combination of both drugs in 4 eyes (15.4%). The volume injected was 0.05 mL in 24 eyes (92.3%) and 0.1 mL in 2 eyes (7.7%). The mean number of injections was 4.5 (range 1–17, median 3) at the last follow-up. The mean number of injections at 6 months, 12 months, and 24 months was, respectively, 2.4 (*n* = 26), 3.2 (*n* = 20), and 5.5 (*n* = 13). CNV was subfoveal in 14 eyes (53.8%), juxtafoveal in 5 eyes (19.2%), extrafoveal in 5 eyes (19.2%), and peripapillary in 2 eyes (7.7%). The mean size of CNV was 1.3 disc diameter (range 0.3–3 disc diameter; *n* = 22 eyes). There was no correlation between the size of the choroidal osteoma and initial visual acuity (Pearson correlation = 0.19; two-tailed probability *P* = 0.36). Eight cases presented with subretinal hemorrhage and the rest with subretinal fluid. Photodynamic therapy (PDT) was given concomitant with initial anti-VEGF injection in 3 eyes (11.5%), repeated twice in 2 eyes (7.7%) and repeated 3 times in one eye (3.8%).

Mean central foveal thickness was 447 microns (*n* = 20 eyes) at baseline, 339 microns (*n* = 20 eyes) at 6 months after intraocular injection, 320 microns (*n* = 11 eyes) at 1 year, and 265 microns (*n* = 9 eyes) at 2 years. Fifteen of 16 eyes showed a decrease in central foveal thickness at 6 months of therapy. By paired comparison, the mean decrease from baseline was 119.7 microns at 6 months (*n* = 15; *P* = 0.001), 105.3 microns at 1 year (*n* = 10; *P* = 0.03), and 157.6 microns at 2 years (*n* = 7; *P* = 0.08). BCVA improved by 3.3 lines at 6 months after therapy (*n* = 26; *P* < 0.001) (20 eyes had improvement in BCVA, 3 had stable vision, and 3 had loss of vision at the 6-month follow-up), 2.8 lines (*n* = 20; *P* = 0.01) at 1 year, and 3.1 lines (*n* = 13; *P* = 0.049) at 2 years. No ocular or systemic adverse events were noted.

To analyze the effect of anti-VEGF alone (without PDT) in more or less acute cases with CNV, we eliminated 2 eyes that had chronic signs of CNV (atrophic thin retina, very large amount of submacular fluid) and 6 eyes that had concomitant PDT, leaving 18 eyes for analysis. Visual acuity improved from 0.77 (6/35 or 20/118) to 0.44 (6/17 or 20/55) at 6 months (*n* = 18) (*P* = 0.006), a gain of 3.3 lines (Table 2). Thirteen eyes (50.0%) had improvement in BCVA, 2 eyes (7.7%) had stable vision, and 3 eyes (11.5%) had loss of vision at the 6-month follow-up. Also, there was visual improvement of 3.4 lines at 1 year (0.67 to 0.34; *n* = 14; *P* = 0.01) and 4.9 lines at 2 years (0.79 to 0.30; *n* = 7; *P* = 0.03). Moreover, by paired comparison, the decrease in central foveal thickness from baseline was 139.5 microns at 6 months (*n* = 11; *P* = 0.002), 123.7 microns at 12 months (*n* = 6; *P* = 0.1), and 196.4 microns at 24 months (*n* = 5; *P* = 0.1). In the other group of concomitant PDT, BCVA improved by 3.9 lines at the 6-month follow-up from 0.65 (6/27 or 20/90) to 0.26 (6/11 or 20/36) (*P* = 0.04) (Table 3). In addition, Table 4 details the characteristics of 13 cases of choroidal osteomas treated with anti-VEGF injections published in the literature.

## 4. Discussion

Visual impairment in eyes with choroidal osteoma can be attributable to several factors including subfoveolar location, foveal edema, photoreceptor degeneration [6], serous retinal detachment, and CNV [3]. Without any therapy, choroidal osteoma-associated CNV can result in a progressive and permanent loss of visual acuity. CNV occurs in 31% to 47% of patients followed for 10 years [3, 6].

The cause for the development of a CNV in eyes with a choroidal osteoma has not been determined, but choroidal osteomas with overlying hemorrhage or irregular surface appeared at higher risk of developing a CNV [3]. Osteomas, in general, have a high bone turnover reflecting their high metabolic rate and hence may steal blood supply from adjacent tissues, especially overlying retinal pigment epithelium which may upregulate the expression of VEGF. Presumably, attenuation and disruption of the retinal pigment epithelium-Bruch membrane complex overlying the choroidal osteoma allows over years for the development of CNV.

There is no standard treatment for a choroidal osteoma. Various treatments for CNV have been tried, but they do not usually halt visual loss. The results of one study showed that photocoagulation of an extrafoveal classic CNV was successful in closing CNV in 25% of treated eyes [2]. However, photocoagulation can stimulate rapid vascular remodeling and anastomoses in choroidal osteoma [3]. Another study reported that the CNV can be surgically removed, but the postoperative visual acuity was poor at 6/95 (20/320) [4]. PDT has been partially successful in treating CNV in eyes with choroidal osteoma. Earlier studies showed that, 6 months after a single PDT, the metamorphopsia can resolve completely; in one study, the visual acuity was not changed [5] and in another study it improved from 6/60 (20/200) to 6/6 (20/20) [6]. Another study reported that four PDT applications led to closure of the CNV, but the final vision was 6/35 (20/120) [7]. PDT has successfully caused closure of extrafoveal CNV in choroidal osteoma [6]. Laser photocoagulation or PDT in choroidal osteomas with or without CNV may carry the risk of decalcification of choroidal osteoma [10]. Shields et al. [10] proposed that photodynamic therapy could be a therapeutic modality for choroidal neovascularization and induction of decalcification of extrafoveal osteoma to prevent tumor growth into the foveola. However, these results should not be extrapolated to subfoveal choroidal osteoma because decalcification of subfoveal choroidal osteoma could result in worse visual acuity because of loss of retinal pigment epithelium and choroidal perfusion [1, 3, 6, 10].

TTT was effective in obstructing the CNV but the visual outcome was also poor. An earlier report indicates that, at 10 months after one TTT application, vision improved from 6/24 (20/80) to 6/18 (20/60), and the vision was maintained with a scarred CNV [8]. In another report, the final visual acuity was 6/60 (20/200) after three TTT applications [9]. Combination therapy of PDT and anti-VEGF injections reduced the number of anti-VEGF injections, hence reducing the risk of vision-threatening complications. The reduction in the number of injections was marginal in the current series (Table 2) and there was little difference between the use of PDT or its omission, although a direct comparison could not be done because of the small number in the current series as well as difference in protocols in administration of both anti-VEGF agents and PDT.

Ahmadieh and Vafi [18] reported the dramatic response of a juxtafoveal CNV associated with choroidal osteoma to a single intravitreal injection of bevacizumab in a 19-year-old female with visual improvement from 6/60 (20/200) to 6/7.5 (20/25) and resolution of metamorphopsia with the treatment effect persisting during a 9-month follow-up period. Subsequently, the positive effects of intravitreal anti-VEGF injections were confirmed in 10 cases with CNV associated with choroidal osteoma (Table 4). Kubota-Taniai et al. [25] described the long-term effect of 2 intravitreal bevacizumab injections (4 months apart) in complete angiographic regression of CNV for a period of 4 years in a 12-year-old girl with visual improvement from 6/30 (20/100) to 6/9 (20/30). We noted similar response in 8 out of 26 eyes needing only 1 to 2 injections with maintenance of visual improvement. It is possible that small osteomas or osteoma that do not grow do not need further injections. This issue was not studied in the current protocol. The growth of the tumor during followup was not assessed also and it could be that growing tumors secrete more VEGF and require more injections. The young age of the patients with osteoma may partly explain the good response to anti-VEGF therapies. A single case had retinal pigment epithelial tear after anti-VEGF injection [29].

## 5. Conclusions

The inherent limitations of this study include its small number, retrospective nature, limited follow-up, lack of a standard therapeutic protocol, lack of a control group, and difference in OCT machines among centers. However, our results suggest that intravitreal bevacizumab or ranibizumab might be an effective therapeutic option for choroidal osteoma-associated CNV that is causing deterioration in vision, particularly when the CNV is juxtafoveolar or subfoveolar in location. In eyes where the CNV is not subfoveolar, adjunctive therapies such as laser photocoagulation or PDT could be considered. Further studies with longer follow-up are indicated to confirm the long-term efficacy of bevacizumab or ranibizumab monotherapy in choroidal osteomas.

## Figures and Tables

**Table 1:** Clinical profile of 26 eyes of 25 cases from the Collaborative Choroidal Osteoma Group*.

Case *N*.	Age	Gender	Osteoma location	CNV location	Initial vision	Final vision	Initial CFT	6-month CFT	Type of injections	Follow-up (month)	Number of injection	Osteoma longest dimension (disc diameter)
1	34	F	Subfoveal	Juxtafoveal	20/300 (6/90)	20/60 (6/18)	NA	174	Bevacizumab	18	2	3

2	37	M	Posterior pole	Subfoveal with blood	20/25 (6/7.5)	20/20 (6/6)	NA	NA	Bevacizumab	52	8	10

3	43	F	Subfoveal	Subfoveal with blood	20/32 (6/9.5)	20/100 (6/30)	294	197	Ranibizumab	18	1	4

4	8	F	Subfoveal	Subfoveal with blood	CF	20/40 (6/12)	434	NA	Bevacizumab	24	2	7

5	28	M	Subfoveal	Subfoveal with SRF	20/60 (6/18)	20/80 (6/24)	373	252	Bevacizumab	46	3	7

6	13	F	Subfoveal	Subfoveal with blood	20/100 (6/30)	20/50 (6/15)	307	212	Ranibizumab	42	3	5

7	27	M	Superotemporal arcade	Extrafoveal with blood	20/80 (6/24)	20/30 (6/9)	NA	NA	Ranibizumab	18	7	9

8	27	F	Subfoveal	Extrafoveal	20/30 (6/9)	20/20 (6/6)	264	207	Bevacizumab	12	1	4

9	46	F	Subfoveal	Subfoveal with blood	20/300 (6/90)	20/80 (6/24)	244	NA	Bevacizumab	60	8	6

10	37	F	Posterior pole	Peripapillary	20/80 (6/24)	20/20 (6/6)	790	360	Bevacizumab	26	4	1

11	28	F	Posterior pole	Peripapillary	20/400 (6/120)	20/400 (6/120)	Highly elevated serous macular detachment	highly elevated serous macular detachment	Ranibizumab	8	2	4

12	48	F	Subfoveal & juxtafoveal	Subfoveal & juxtafoveal	20/40 (6/12)	20/20 (6/6)	298	306	Ranibizumab number 5, Bevacizumab number 10	35	15	3

13	25	F	Subfoveal	Subfoveal	20/80 (6/24)	20/20 (6/6)	203	166	Ranibizumab	15	1	1

14	33	F	Subfoveal	Subfoveal	(20/67) 6/20	(20/320) 6/95	316		Bevacizumab	24	3	2

15	22	M	Subfoveal	Juxtafoveal	CF 1 m	CF 2 m	NA	675	Bevacizumab	7	3	6

16	41	F	Juxtafoveal	Juxtafoveal	20/200 (6/60)	20/200 (6/60)	427	258	Ranibizumab number 3 Bevacizumab number 1	6	4	3

17	24	M	Extrafoveal	Extrafoveal with blood	20/60 (6/18)	20/30 (6/9)	350	210	Bevacizumab	54	17	2

18	14	M	Subfoveal	Subfoveal with blood	CFNF	CFNF	>1000	>500	Bevacizumab number 4 Ranibizumab number 5	26	9	8

19	35	F	Posterior pole	Peripapillary	20/70 (6/21)	20/40 (6/12)	600	400	Bevacizumab	12	2	7

20	45	M	Subfoveal	Juxtafoveal	20/63 (6/19)	20/32 (6/9.5)	237	222	Bevacizumab	6	2	1.2

21	13	F	Subfoveal	Subfoveal	20/200 (6/60)	20/63 (6/19)	339	249	Bevacizumab	6	2	4

22	20	M	Subfoveal	Juxtafoveal	20/70 (6/21)	20/40 (6/12)	282	171	Bevacizumab	71	1	4

23	22	M	Subfoveal	Subfoveal	20/160 (6/48)	20/250 (6/75)	427	225	Bevacizumab	46	5	4.9

24	11	M	Juxtafoveal	Extrafoveal	20/20 (6/6)	20/20 (6/6)	NA	NA	Bevacizumab	16	3	3.4

25	57	F	Subfoveal	Subfoveal	20/800 (6/240)	20/30 (6/9)	NA	283	Ranibizumab number 3 Bevacizumab number 3	21	6	4

26	25	F	Extrafoveal	Subfoveal	20/300 (6/90)	20/100 (6/30)	360	320	Bevacizumab	12	2	6

*N = number; CFT = central foveal thickness; SRF = subretinal fluid; F = female; M = male; NA = not available; CF = counting fingers; CFNF = finger counting at near face.

**Table 2:** Mean lines of visual acuity improvement after anti-VEGF injections (excluding 6 PDT & 2 chronic CNV eyes)*.

	Initial (preinjection)	6 months	12 months	18 months	24 months
Number of eyes	18	18	14	10	7
Mean line of improvement	0.77	3.3	3.4	3.2	4.9
Paired comparison (*P* value)		0.006	0.01	0.03	0.03

*VEGF = vascular endothelial growth factor; CNV = choroidal neovascularization; PDT = photodynamic therapy.

**Table 3:** Summary profile comparing patients who underwent PDT plus anti-VEGF to patients who had anti-VEGF therapy alone (excluding chronic cases of CNV) at the 6-month follow-up*.

Category	Age	Gender (M/F)	Mean follow-up	CNV location	Initial vision (logMar)	Final vision (logMar)	Number of PDT sessions	Number of injections
PDT group *n* = 6	27	2/4	32 months	Subfoveal 5 Juxtafoveal 1	0.65	0.40	1.8 (1–4)	3.9

No PDT group *n* = 18	32	7/11	24 months	Subfoveal 8 Juxtafoveal 4 Extrafoveal 4 Peripapillary 2	0.77	0.42	0	4.4

*VEGF = vascular endothelial growth factor; CNV = choroidal neovascularization; M = male; F = female; PDT = photodynamic therapy; logMar = logarithm of the minimum angle of resolution.

**Table 4:** Literature review of 13 choroidal new vessels associated with choroidal osteomas treated with intravitreal anti-VEGF injections*.

Author	Age	Gender	Osteoma location & longest dimension	CNV location	Initial vision	Final vision	Initial CFT (microns)	Final CFT (microns)	Type & number of injections	Follow-up (month)
Ahmadieh and Vafi [18]	19	F	Juxtafoveal 3DD OD	Juxtafoveal	6/60	6/7.5	544	240	One 1.25 mg bevacizumab	9 (regression of CNV)

Narayanan and Shah [19]	25	F	Peripapillary OS	Subfoveal	CF 1.5 m	6/35	NM	NM	Two 1.25 mg bevacizumab	4 (regression of CNV)

Shields et al. [20]	34	F	Subfoveal 7DD OD	Subfoveal	6/30	6/9	NM	NM	One 1.25 mg bevacizumab & three 0.5 mg ranibizumab	6 (fibrotic CNV with flat macula)

Song et al. [21]	24	M	Peripapillary 2.5DD	Juxtafoveal	CF 20 cm	6/60	NM	NM	Two 1.25 mg bevacizumab	10; prior to PDT

Song and Roh [22]	43	F	Subfoveal 4DD OS	Subfoveal	6/60	6/30	NM	NM	One 0.5 mg ranibizumab	6 (regression of CNV)

Rao and Gentile [23]	24	M	Subfoveal 2DD OS	Subfoveal	6/18	6/9	NM	NM	Three 1.25 mg bevacizumab	5

Ayachit et al. [24]	27	F	Peripapillary 3DD OD	Juxtafoveal	6/7.5	6/6	NM	NM	One bevacizumab (dose NM)	6 (regression of CNV)

Kubota-Taniai et al. [25]	12	F	Peripapillary 3DD OS	Extrafoveal	6/30	6/9	NM	NM	Two 1.25 mg bevacizumab	48 (regression of CNV)

Salehipour et al. [26]	19	F	NM	NM	NM	NM	NM	NM	Two 1.25 mg bevacizumab	7

Wu et al. [27]	46	F	Subfoveal OS	Subfoveal	6/30	6/12	NM	116 micron flattening	Two 1.25 mg bevacizumab	23

Wu et al. [27]	57	F	Peripapillary 10DD OD	Subfoveal	6/240	6/9	NM	NM	Three 0.5 mg ranibizumab	14 (fibrosis of CNV)

Carle et al. [28]	20	M	Macular OD	Occult	6/24	6/12	NM	NM	Six 1.25 mg bevacizumab	13

Erol et al. [29]	28	F	Peripapillary and macular 4DD OD	Subfoveal	6/120	6/24 after first injection and then 6/60 after second injection	NM	NM	Two 0.5 mg ranibizumab	2 (Tear of the retinal pigment epithelium after ranibizumab injection)

*VEGF = vascular endothelial growth factor; CNV = choroidal neovascularization; CFT = central foveal thickness in microns; DD = disc diameter; NM = not mentioned; OD = right eye; OS = left eye; M = male; F = female; PDT = photodynamic therapy.
